# A review of clopidogrel resistance in lower extremity arterial disease

**DOI:** 10.1016/j.jvsvi.2024.100112

**Published:** 2024

**Authors:** Kerry A. Burke, John H. McDermott, Stuart J. Wright, William G. Newman, Nicholas S. Greaves

**Affiliations:** a Manchester Centre for Genomic Medicine, St Mary’s Hospital, Manchester University NHS Foundation Trust, Manchester, United Kingdom; b Division of Evolution, Infection, and Genomics, School of Biological Sciences, University of Manchester, Manchester, United Kingdom; c Manchester Vascular Centre, Manchester Royal Infirmary, Manchester University NHS Foundation Trust, Manchester, United Kingdom; d Manchester Centre for Health Economics, The University of Manchester, Manchester, United Kingdom

**Keywords:** Clopidogrel, Clopidogrel resistance, Vascular surgery

## Abstract

**Objective:**

Lower extremity arterial disease (LEAD) is a prevalent condition that produces a significant burden on health care systems. Patients with LEAD have an increased risk of major adverse cardiovascular events as well as major adverse limb events. Despite significant variation in guidance on antiplatelet therapy for LEAD worldwide, many governing bodies recommend clopidogrel as the preferred single anti-platelet agent. Clopidogrel is also used frequently in post-revascularization regimens, either as a single agent or as part of dual antiplatelet therapy. Clopidogrel is a thienopyridine prodrug that is metabolized in the liver by the CYP2C19 enzyme. Genetic variations in *CYP2C19* are common and can influence an individual’s ability to metabolize clopidogrel to its active metabolite.

**Methods:**

This work completes a narrative review of the literature to consider whether *CYP2C19* genetic testing should be routinely implemented in patients who are to be prescribed clopidogrel to improve outcomes in patients with LEAD.

**Results:**

Recent advances in both cardiac and stroke medicine have demonstrated a role for patient genotyping to identify poor clopidogrel metabolizers and adopt alternative therapeutic strategies in these patient groups. This approach has been shown to improve clinical outcomes and has been incorporated into national and international guidance. Research studies suggest an association between *CYP2C19* loss of function alleles and adverse outcomes in patients with LEAD taking clopidogrel.

**Conclusions:**

The introduction of a precision medicine strategy in vascular surgery may have the potential to significantly improve clinical outcomes in this complex group of patients with multiple comorbidities.

Lower extremity arterial disease (LEAD) is a prevalent condition, estimated to affect over 113.4 million people globally.^1^ Due to concomitant systemic atherosclerosis, patients with LEAD have an increased risk of major adverse cardiovascular events (MACE), including myocardial infarction, cerebrovascular events, and vascular death. Studies have demonstrated a six-fold increased risk in cardiovascular death in patients with LEAD.^2^ Chronic limb-threatening ischemia (CLTI) is associated with a significant risk of major adverse limb events (MALE), including loss of vessel patency, need for surgical intervention, and major amputation.^3^ Patients with CLTI have the most severe degree of arterial disease, and thus have the highest risk of MACE, with a significant reduction in life expectancy. In the recent BASIL-2 trial, an interventional trial investigating revascularization strategies in patients with CLTI, the median survival time was only 3.8 years.^4^

All major guidelines recommend single antiplatelet therapy for patients with symptomatic LEAD in the form of clopidogrel,^5^^,^^6^ or the choice of clopidogrel or aspirin,^7^, ^8^, ^9^ to significantly reduce the risk of MACE. Clopidogrel was initially shown to be more effective than aspirin in reducing MACE with fewer bleeding events in the CAPRIE study.^10^ Subsequent meta-analyses have also demonstrated a significant reduction in MACE in patients with LEAD on clopidogrel compared with aspirin.^11^^,^^12^ The American Heart Association also recommends considering dual antiplatelet therapy (DAPT) in patients with LEAD who are at high risk of MACE and at low risk of bleeding.^7^

Once a patient has undergone a revascularization procedure, the antiplatelet strategy has the additional role of maintaining vessel, graft, or stent patency. Thickening of the arterial intimal layer, also known as neo-intimal hyperplasia, occurs in response to surgical trauma and can result in arterial, graft, or stent lumen stenosis or occlusion.^13^ Some guidelines recommend long-term single antiplatelet therapy after surgical or endovascular revascularization,^9^ or after prosthetic bypass.^6^ A number of guidelines also advocate the use of DAPT for a period of time after certain endovascular procedures such as stenting,^6^, ^7^, ^8^, ^9^ after it was shown to significantly reduce target-lesion revascularisation,^14^ reduce platelet activation, and improve functional outcomes.^15^ DAPT is also recommended following prosthetic bypass^7^, ^8^, ^9^ after a pre-specified sub-group analysis in the CASPAR study demonstrated a significant reduction in MACE and death.^16^

A lack of large prospective trials directly comparing these different therapy regimens has resulted in significant variation in the guidance on antiplatelet therapy for LEAD worldwide. However, many governing bodies recommend clopidogrel as the preferred single anti-platelet agent. Clopidogrel use also features heavily in post-procedure therapy regimens, either as a single agent or as part of DAPT. Despite this, there are significant variations in its pharmacological effectiveness based upon genetic factors, which can be identified by genetic testing.

Clopidogrel is a thienopyridine prodrug that is absorbed in the intestines through the ABCB1 transporter. It is converted from an inactive to active metabolite through two oxidative steps in the liver (Fig).^17^ This active metabolite irreversibly binds to the platelet receptor P2Y12, preventing platelet activation and aggregation.^18^ The hepatic biotransformation of the clopidogrel pro-drug requires several cytochrome P450 (CYP450) enzymes, of which the CYP450 subfamily C member 19 (CYP2C19) has the most significant role.

**Fig:**
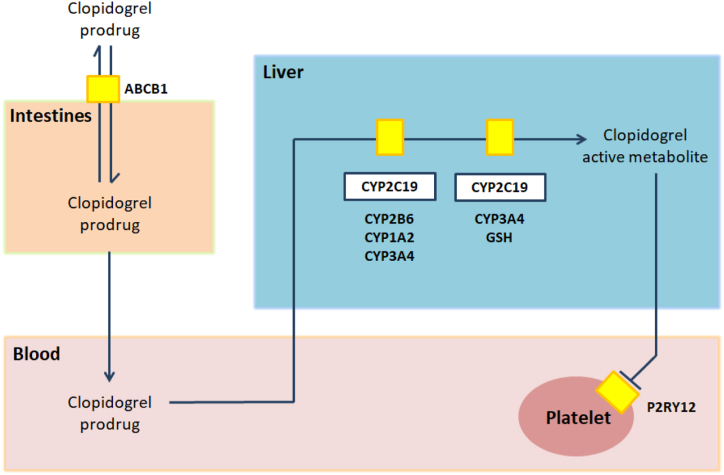
Clopidogrel metabolism.

The *CYP2C19* gene is highly polymorphic and has over 35 allele haplotypes.^18^ Clopidogrel metabolizer status is determined by an individual’s allele diplotype, with the ‘wild type’ allele ∗1 known as the normal metabolizer (NM).^17^ The most common ‘loss of function’ (LoF) alleles are ∗2, followed by ∗3. Intermediate metabolizer (IM) status is defined as the heterozygous state, where an individual carries one wild-type allele and one LoF allele. Poor metabolizer (PM) status is when an individual has biallelic variants, with two LoF alleles.

The estimated prevalence of each *CYP2C19* allele varies depending on ethnicity (Table). The wild type allele ∗1 is found in around 63% of European populations.^19^ LoF allele ∗2 is estimated to be present in around 27% of Central/South Asians, 28% of East Asians, 16% of sub-Saharan Africans, and 15% of Europeans. LoF allele ∗3 is seen less frequently, with an estimated prevalence of 7% in East Asians. There are a number of other LoF alleles, including ∗4, ∗5, ∗6, and ∗7, but their individual prevalences are less than 1%.^19^

**Table:** Estimated *CYP2C19* allele and diplotype prevalence according to ethnicity

	European	American	Central/South Asian	East Asian	Sub-Saharan African	Oceanian
*CYP2C19* allele						
1∗	63	79	54	60	55	19
2∗	15	12	27	28	16	61
3∗	<1	<1	2	7	<1	15
*CYP2C19* diplotype						
NM	40	63	30	38	37	4
IM	26	21	41	46	30	37
PM	2	1	8	13	4	57

*IM*, Intermediate metabolizer; *NM*, normal metabolizer; *PM*, poor metabolizer.

Data are presented as percentages.

1∗ is the wild-type allele; 2∗ and 3∗ are loss of function (LoF) alleles.

Adapted from CYP2C19 frequency table.^38^

It is the combination of these alleles in diplotype that determines a patient’s clopidogrel metabolizer status (Table).^20^ In a large cohort of British Bangladeshi and Pakistani ancestry participants, 57% were found to have at least one LoF *CYP2C19* allele, and 13% had two LoF alleles, suggesting that LoF frequency in this population is likely to be higher than previously estimated.^21^ The presence of *CYP2C19* LoF alleles has been shown to result in high residual platelet reactivity, lower levels of active metabolite, and reduced platelet inhibition.^22^ Increasing the dose of clopidogrel has not been shown to provide adequate platelet inhibition in patients who are PM.^23^ An individual’s pharmacogenetics profile may therefore dramatically affect clopidogrel metabolism and the clinical effectiveness of the drug. This work completes a narrative review of the literature to consider whether *CYP2C19* genetic testing should be routinely implemented in patients who are to be prescribed clopidogrel to improve outcomes in patients with LEAD.

## CYP2C19 genetic testing in clinical practice

The United States Food and Drug Administration issued a boxed warning on clopidogrel in 2010 to warn that patients who are *CYP2C19* PM will experience reduced drug effectiveness in preventing MACE.^24^ Since then, the role of patient *CYP2C19* genotyping has gathered momentum in both cardiac and stroke medicine. Although ticagrelor and prasugrel feature prominently in the management of acute coronary syndrome, clopidogrel still maintains a key role including in patients with stable coronary artery disease requiring percutaneous coronary intervention (PCI).^25^^,^^26^ Patients undergoing PCI with *CYP2C19* LoF alleles who are treated with clopidogrel have been shown to be at a significantly increased risk of MACE, including coronary stent thrombosis.^27^, ^28^, ^29^ A number of studies have demonstrated the benefits of genotype-guided therapy for patients undergoing PCI.^30^^,^^31^ Genotype-guided testing was also found to be cost-effective when compared with standard ticagrelor or prasugrel treatment, with a hypothetical cohort of 1000 patients gaining 8.98 quality-adjusted-life-years with cost savings of €725,551 (€10,650,062 vs €11,375,613, respectively).^32^ The analysis considered both generic and non-generic costings for ticagrelor and prasugrel. The importance of genotype-guided therapy is now reflected in a number of major guidelines, with recommendations to provide alternative medication in *CYP2C19* LoF carriers in PCI.^18^^,^^24^ The American Heart Association states that genetic testing can be considered in select high-risk patients undergoing PCI,^33^ whereas the French National Network of Pharmacogenetics (RNPGx) and the Royal Dutch Pharmacists Association Pharmacogenetics Working Group now recommend routine *CYP2C19* genotyping prior to PCI.^34^^,^^35^

Clopidogrel is also used to treat patients with ischemic stroke to reduce their risk of recurrent events. There is now convincing research that *CYP2C19* LoF carriers who have had a stroke are at a significantly higher risk of MACE when treated with clopidogrel.^36^, ^37^, ^38^ An early cost-effectiveness analysis demonstrated that routine patient genotyping after ischemic stroke could provide significant health and cost gains by preventing recurrent stroke.^39^ At present, both the Royal Dutch Pharmacists Association Pharmacogenetics Working Group and draft guidance from the National Institute for Health and Care Excellence in England recommend routine *CYP2C19* genotyping in patients who have had a stroke.^34^^,^^40^ There are therefore now established and evolving roles for routine *CYP2C19* genotyping in both cardiac and stroke medicine across the globe.

## CPY2C19 variants in lower extremity arterial disease

Unlike in cardiac and stroke medicine, few studies have investigated the implications of *CYP2C19* variants on clinical outcome in patients with LEAD. The current literature is characterized by small single-center observational studies from different demographic populations. The largest study was undertaken by Lee et al in 2019 from a single center in Taiwan. The authors retrospectively studied 278 patients taking clopidogrel who had undergone endovascular revascularization for CLTI.^41^ They found amputation-free survival at 12 months to be significantly lower in PM and IM patients compared with EM patients (56.6%, 66.1%, and 82.1%, respectively; *P* < .0001). All-cause mortality at 12 months was also higher in PM and IM patients compared with EM patients (71.3%, 72.2%, and 83.7%, respectively; *P* = .007). LoF alleles were shown to be independent predictors of amputation and all-cause mortality.^41^ They concluded that *CYP2C19* genetic variants can significantly influence both amputation-free and all-cause mortality in patients with CLTI taking clopidogrel after endovascular intervention.

The only prospective study to investigate the impact of LoF alleles in LEAD was by Guo et al in China in 2014, which included 120 patients with LEAD in the superficial femoral artery requiring endovascular revascularization.^42^ Patients were started on DAPT for 12 months post-procedure with regular imaging of stent patency. The primary outcome was in-stent restenosis or occlusion. Only 50 patients were included in the final analysis as the authors excluded patients who did not undergo successful angioplasty with stenting, and there was also significant loss to follow-up. Patients with one LoF allele had a significantly higher rate of ischemic events compared with patients without (59.0% vs 20.8%; *P* = .008). This finding was even more pronounced in patients with two LoF alleles compared with patients without (100% vs 20.8%; *P* = .002). The 1-year primary patency rate was also lower in LoF carriers (*P* = .006). Of those patients included in the final analysis, 60% had intermittent claudication. Although the generalizability of this study is limited by its single-center design and small sample size, the results suggest an association between *CYP2C19* LoF alleles and poor patient outcomes after endovascular stent therapy, despite the concomitant use of aspirin.

Diaz-Villamarin et al undertook a retrospective single-center study in Spain in 2016 to investigate the effects of *CYP2C19* and *ABCB1* genotype on clinical outcome in LEAD.^43^ They included 72 patients who had undergone angioplasty and were taking clopidogrel, the majority of whom had claudication (86%). Their primary outcome was target lesion restenosis or occlusion at 12 months post-angioplasty. They found that *CYP2C19* LoF ∗2 was associated with a significantly higher risk of target lesion restenosis over 12 months (odds ratio [OR], 5.0; 95% confidence interval [CI], 1.75-14.25). There was no difference in outcome reported in *CYP2C19* LoF carriers taking clopidogrel compared with those taking DAPT. The duration of clopidogrel therapy was also variable in the study. A meta-analysis of their results along with those of Guo et al^42^ demonstrated a significantly increased rate of new ischaemic events in patients with *CYP2C19* ∗2 (pooled OR, 5.4; 95% CI, 2.3-12.7).^43^

Huang et al undertook a systematic review to identify the association between *CYP2C19* LoF alleles and clinical outcomes in LEAD in patients taking clopidogrel.^44^ The authors included the above three observational studies, and a fourth study by Pastromas et al,^45^ which investigated outcomes based on platelet function rather than genetic testing. The primary outcome was clinical effectiveness, which included clinical non-response, amputation, restenosis or occlusion, ischemic events, and target limb reintervention. All the studies demonstrated a higher risk of reduced clinical effectiveness of clopidogrel in patients with a *CYP2C19* LoF allele. However, due to significant differences in study design and outcomes, as well as small sample sizes, the authors were unable to calculate a pooled OR to support this finding.^44^

This meta-analysis by Huang et al highlights the sparsity of research available to determine the implications of *CYP2C19* variants on outcomes in LEAD.^44^ The studies described all have methodological limitations and are at risk of bias. They are all based on single-center data, small sample sizes, and mostly retrospective design. Importantly, none of the studies have included MACE in their primary outcome, prevention of which is an important role of clopidogrel use in this population. All the studies have focused on outcomes for patients who have had endovascular revascularization alone, excluding the large group of patients who undergo open surgery or hybrid procedures. The studies also include a significant proportion of patients with intermittent claudication. This is a patient group for which major guidelines advocate best medical therapy and a supervised exercise program in the first instance, with revascularization only considered in select patients.^3^^,^^7^^,^^46^ This is because early revascularization in patients with intermittent claudication has been shown to increase the 5-year risk of major amputation as well as the need for reintervention.^47^ Conversely, revascularization should be considered in all patients with CLTI due to their significantly increased risk of limb loss. This is, therefore, an essential patient group to include in these clinical studies.

Despite these limitations, these studies do however consistently demonstrate a significant association between *CYP2C19* LoF alleles and poor clinical outcomes after endovascular revascularization in LEAD. This is in keeping with the findings from studies in both cardiac and stroke medicine.

## High on-treatment platelet reactivity

High on-treatment platelet reactivity (HTPR) describes the finding of normalized platelet function despite being on anti-platelet therapy.^48^ *CYP2C19* LoF alleles have been shown to result in HTPR,^41^^,^^42^ but there are many non-genetic factors that can influence platelet function. This can create a mismatch between patient genotype and actual metabolizer status, a phenomenon termed phenoconversion.^49^

Platelet function is affected by a range of disease states associated with LEAD. The association between diabetes and reduced clopidogrel responsiveness has been highlighted in a number of studies.^45^^,^^50^^,^^51^ Diabetic patients on clopidogrel therapy have been found to have significantly lower levels of clopidogrel active metabolite^50^ and increased platelet reactivity.^51^ Chronic kidney disease is also independently associated with clopidogrel resistance, with an estimated 50% to 80% of patients with end-stage renal failure being found to have high residual platelet reactivity while on clopidogrel therapy.^52^ This may be explained by the increased platelet turnover rate, reduced drug bioavailability, and clotting dysfunction seen in chronic renal failure.^53^ There is also an association between clopidogrel non-response and CLTI, which may also be explained by increased platelet reactivity.^45^ The presence of CLTI is known to result in a pro-thrombotic state, with significantly increased P-selectin levels.^54^

The interaction of other medications has also been shown to reduce the activation of clopidogrel. The concomitant use of omeprazole/esomeprazole with clopidogrel has been shown to increase the rate of MACE regardless of LoF status, due to direct inhibition of *CYP2C19*.^55^ Other factors influencing platelet function include obesity, gender, smoking, and patient medication compliance.^56^

HTPR has been estimated to be present in over 50% of patients with LEAD, which is higher than the incidence seen in cardiac and stroke patient cohorts.^45^^,^^57^ The exact reason for this is unclear, but it may reflect differences in the prevalence and severity of comorbidities as well as patient polypharmacy. Studies that have investigated the impact of HTPR on clinical outcomes in LEAD do not show a consistent effect. In the previously described studies, both Lee et al^41^ and Guo et al^42^ demonstrated *CYP2C19* LoF carriers to have a reduced response to clopidogrel on their platelet function testing (*P* < .001 and *P* = .022, respectively). However in both studies, unlike with LoF allele status, HTPR was not independently associated with worse clinical outcome. Other work has also shown similar rates of target lesion revascularization, MALE, and MACE in patients with HTPR compared with those without, after endovascular intervention.^58^^,^^59^ In contrast, several studies have demonstrated significantly increased rates of reintervention,^45^^,^^60^ MACE,^57^ MALE,^48^^,^^57^^,^^61^ and death^62^ in patients with clopidogrel HTPR undergoing endovascular procedures.

Due to the multiple factors influencing platelet function, HTPR cannot be used as a direct marker of *CYP2C19* LoF allele status. The variation in study results may be in part due to the different diagnostic platelet function testing used. There were also variable thresholds at which platelet function was defined as HTPR. Furthermore, the optimum timing of platelet testing is also unknown, with evidence showing that platelet function may develop in a time-dependent fashion and may need testing at multiple time points.^63^^,^^64^ This is likely to make such a strategy more cumbersome to operationalize in a clinical context.

## Discussion

The current evidence body is sparse but does suggest an association between *CYP2C19* LoF alleles and poor outcomes in patients with LEAD taking clopidogrel. Understanding clopidogrel pharmacogenetics could allow medical treatment to be tailored to the individual. If a patient with LEAD was discovered to have *CYP2C19* LoF alleles through genetic testing, this could prompt alternative therapeutic strategies to clopidogrel as first-line therapy. Depending on the patient’s clinical symptoms and revascularization status, this could be in the form of aspirin alone, low-dose rivaroxaban with aspirin, or a vitamin K antagonist. With a condition as prevalent as LEAD, even a modest improvement in treatment outcome could have a dramatic effect on optimizing both clinical outcomes and health care cost savings.

There are different ways to determine patient genotype. Traditionally, this has been done using blood or saliva sample transported to a laboratory for analysis. Rapid point-of-care genotyping testing is now being developed, such as the prototype developed by Genedrive PLC.^65^ This provides genotype results in an hour from a buccal swab, and the analyzer can be placed in the clinic or ward environment. This has the advantage of avoiding the need for blood sampling, sample transporting, and laboratory testing, allowing clinicians to make important medication decisions in the immediate clinical environment.

The timing of testing would also need to be considered. Pre-emptive genotyping of all patients with LEAD at initial presentation would allow for early patient medication optimization but a greater testing burden. Alternatively, a reactive testing strategy could focus on patients with CLTI undergoing revascularization, with genotyping performed in the peri-procedural period. This would provide an opportunity to provide personalized medical therapy to the patient group at highest risk of both MACE and MALE, with a lesser testing burden. The optimal approach to testing has not been formally studied.

The development of a testing strategy would need a consideration of practitioner education, the approach used to testing, the timing of the test, and the integration of genetic information into the patient’s records. There also needs to be a clear pathway to follow for the prescriber once they receive a genetic result. An implementation science method study could provide information around the practicalities of each testing strategy in clinical practice.

Both clopidogrel and aspirin currently offer a significantly cheaper alternative to rivaroxaban, with a lower bleeding risk.^66^^,^^67^ This cost-balance is, however, likely to shift when rivaroxaban loses its patent in 2026 and becomes a generic drug.^68^ Implementing a genetic testing strategy for patients with LEAD would require a formal economic evaluation to estimate the likely costs, benefits, and cost-effectiveness of the strategy. Clopidogrel currently has an evidence-based role in post-procedure therapy regimens in vascular surgery, either as a single agent or as part of DAPT. Studies are needed to directly compare the effectiveness of a genotype-guided clopidogrel therapy regimen against rivaroxaban and aspirin combination therapy in reducing MALE and MACE after both endovascular and open surgical procedures. An effective genotype-led antiplatelet regime may have the potential to reduce reintervention rates, length of inpatient stay, and hospital resource use, and improve patient quality of life. Larger-scale prospective studies are needed to conclusively determine the association between *CYP2C19* LoF alleles and clinical outcome in this patient group to inform a formal cost-benefit analysis. Whether HTPR confers additional prognostic information in addition to the limited genotyping studies available is unclear, but these non-genetic factors are important confounders to consider in genotyping studies.

## Conclusion

Research considering clopidogrel pharmacogenetics in patients with LEAD is small and has methodological limitations but does consistently suggest an association between *CYP2C19* LoF alleles and adverse outcomes in patients with LEAD taking clopidogrel. The introduction of a precision medicine strategy in vascular surgery may have the potential to significantly improve clinical outcomes in this complex group of patients with multiple comorbidities.

## Author Contributions

Conception and design: KB, JM, SW, NG, WN

Analysis and interpretation: Not applicable

Data collection: Not applicable

Writing the article: KB

Critical revision of the article: KB, JM, SW, NG, WN

Final approval of the article: KB, JM, SW, NG, WN

Statistical analysis: Not applicable

Obtained funding: Not applicable

Overall responsibility: WN

## Disclosures

J.M. and W.N. are co-founders of Fava Health.
